# Case Report: Early diagnostic precision matters: navigating therapeutic uncertainty in pediatric BPDCN illustrated by misdiagnosis sequelae and fleeting CAR-T response

**DOI:** 10.3389/fped.2025.1710591

**Published:** 2026-01-08

**Authors:** XiaoYan Chen, JiaYi Wang, WenGe Hao, YuHua Qu, Xiaojing Wang, Hua Jiang, WeiNa Zhang

**Affiliations:** Department of Hematology and Oncology, Guangzhou Women and Children’s Medical Center, Guangzhou Medical University, Guangzhou, China

**Keywords:** BPDCN, CAR-T, CD123, CLL1, diagnosis

Blastic plasmacytoid dendritic cell neoplasm (BPDCN) is a rare hematologic malignancy in children. Phenotypically distinct from other myeloid neoplasms, BPDCN arises from the aberrant transformation of plasmacytoid dendritic cells (pDCs). It typically involves the skin, bone marrow, lymph nodes, and central nervous system (CNS), and is associated with poor prognosis. This disease predominantly affects elderly individuals, with a low incidence in children. Immunophenotypic analysis using multicolor flow cytometry (MFC) is essential for diagnosis, demonstrating pDC-associated markers such as CD123, BDCA2, and TCF4, on BPDCN cells. This aids in the differential diagnosis from conditions like acute myeloid leukemia (AML) with cutaneous involvement (1). Characteristic molecular feature include rearrangements involving the *MYC* or *MYB* genes, which are generally mutually exclusive. *MYB* rearrangements are more common in children and young adults (2). *MYB* plays a role in cell cycle regulation, and its rearrangement can impact the activity of the *MYB* transcription factor. This subsequently disrupts the cell cycle process in pDC progenitors and inhibits their differentiation (3). Due to the rarity of BPDCN and challenges in its pathological definition and diagnosis, standardized prognostic factors for routine clinical practice have not been established. The optimal timing for allogeneic hematopoietic stem cell transplantation (allo-HSCT) remains undefined. Studies have reported improved outcomes with high-risk acute lymphoblastic leukemia (ALL)-like chemotherapy regimens, and allo-HSCT has been utilized in children with multiorgan involvement, positive minimal residual disease (MRD), and/or persistent disease. Promising results have emerged from clinical trials evaluating novel targeted therapies based on the characteristic overexpression of BCL-2 and CD123, including venetoclax and agents such as tagraxofusp (4). The use of CD123-directed chimeric antigen receptor T-cell (CAR-T) immunotherapy to eradicate tumor cells has also been reported, although available data remain limited due to small patient numbers and restricted efficacy to date (5). Thus, the diagnosis and management of this rare disease continue to pose significant challenges.

Herein, we summarize the clinical courses of two pediatric BPDCN patients treated with different therapeutic regimens, who achieved differential treatment outcomes. This report represents the first documentation of BPDCN's variable sensitivity to multiple pharmacological agents. Notably, we identified a novel MYB gene rearrangement (*MYB::TBC1D5*), expanding the molecular spectrum of pediatric BPDCN. We also report the potential utility of AML-type chemotherapy regimens and CLL1-targeted CAR-T therapy in managing this disease.

## Case 1

**Figure 1:**
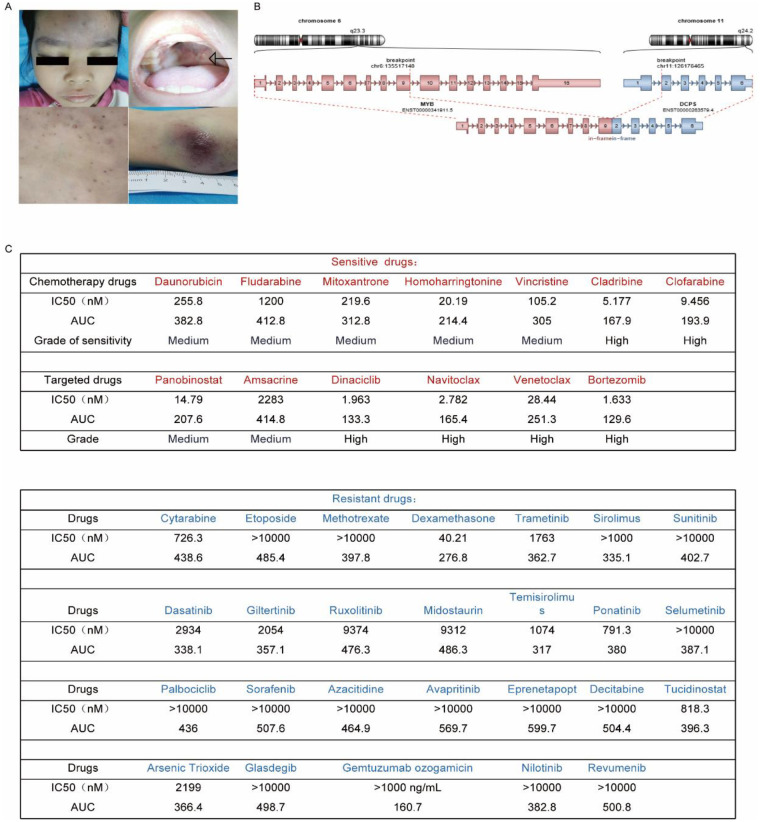
The condition of BPDCN patients treated with the ALL regimen. **(A)** Clinical manifestations before treatment. **(B)** Schematic diagram of *MYB::DCPS* gene fusion. **(C)** *in vitro* chemosensitivity and targeted drugs sensitivity results of the tumor cells from BPDCN patients.

An 8-year-old girl presented with a 4-month history of generalized scattered rash and a 5-day history of intermittent fever. Initial complete blood count revealed leukopenia (WBC 1.93 × 10⁹/L), neutropenia (ANC 0.48 × 10⁹/L), mild anemia (hemoglobin 118 g/L), and thrombocytopenia (platelets 108 × 10⁹/L). Physical examination showed generalized scattered rash, enlarged superficial lymph nodes (e.g., inguinal region), and splenomegaly (Figure 1A). Bone marrow (BM) morphology was consistent with acute leukemia. Immunophenotyping suggested BPDCN (HLA-DR, CD123, CD56, CD17, CD36, CD71, CD4, CD5, CD7, and CD304+). Molecular genetic testing identified *MYB::DCPS* fusion (Figure 1B), *CDKN2A::MTAP* fusion, and mutations in *PTPN1* and *NRAS*. The genetic alterations were identified using RNA sequencing of bone marrow samples. BM histopathology raised suspicion of myeloid leukemia. PET/CT demonstrated multiple enlarged lymph nodes and subcutaneous nodules, concerning for lymphomatous infiltration. Cerebrospinal fluid (CSF) analysis by flow cytometry revealed 55% lymphocytes, of which 45% were abnormal cells expressing HLA-DR, CD4, CD5, CD123, and CD304. Based on the 2024 NCCN guidelines, the diagnosis of BPDCN with involvement of bone marrow, skin, lymph nodes, and CNS was confirmed. The patient received the CCCG-ALL-2020 (6) chemotherapy regimen plus six doses of intrathecal therapy. MRD by flow cytometry in BM, fusion transcripts (*MYB::DCPS*, *CDKN2A::MTAP*), and CSF involvement all turned negative on day 19 and day 46 of induction therapy and following consolidation. She subsequently underwent allo-HSCT with a combined graft from her father (5/10 HLA match) and umbilical cord blood (8/10 HLA match). Complete donor chimerism was achieved by day 12 following the transplant.

This case demonstrates the efficacy of an ALL-directed chemotherapy regimen in achieving rapid multicompartmental remission, facilitating successful allo-HSCT in pediatric BPDCN. It highlights the diagnostic value of comprehensive molecular profiling and CSF flow cytometry. *In vitro* drug sensitivity testing (7) (Figure 1C) identified potential targeted therapeutic options, providing a reference for novel treatments. Current therapeutic approaches for BPDCN include multiple non-mutually exclusive options. However, standardized high-risk stratification remains undefined, and optimal patient selection or timing for HSCT requires further study (8). Beyond conventional therapy, novel agents targeting pathways such as CD123 or BCL-2 hold significant promise, based on biological rationale and drug sensitivity profiling. Comprehensive *in vitro* drug sensitivity profiling in BPDCN is rarely reported, particularly in pediatric cases. Our data provide a preliminary map of potential therapeutic vulnerabilities of BPDCN. The response to venetoclax observed in this case aligns with emerging evidence of BCL-2 dependency in BPDCN. Venetoclax, a BCL2 inhibitor, often in combination with hypomethylating agents, has shown promising activity in BPDCN (9, 10). However, predictors of response and mechanisms of resistance in pediatric BPDCN warrant further investigation.

## Case 2

**Figure 2:**
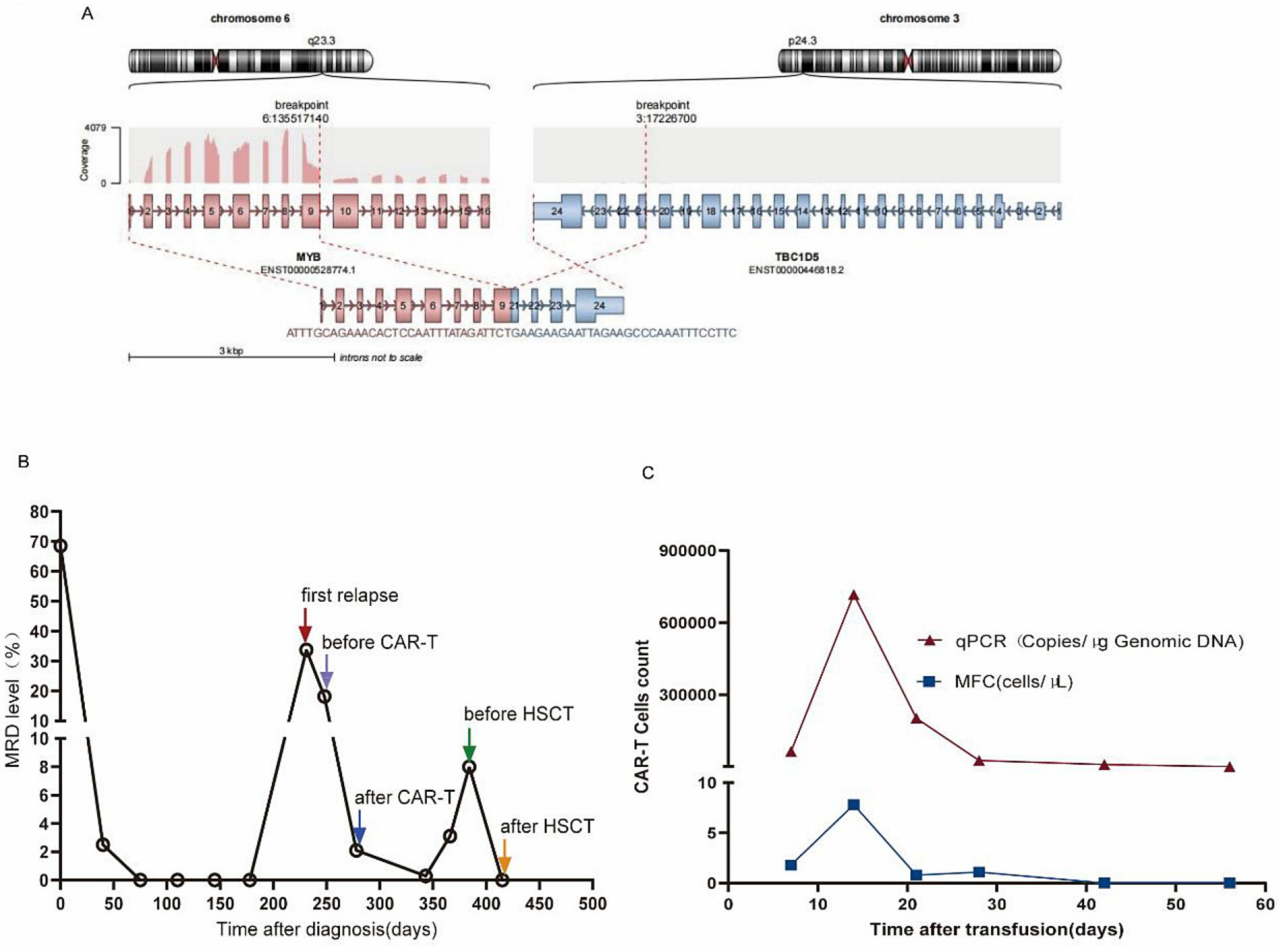
The condition of BPDCN patients treated with AML protocol. **(A)** Schematic diagram of the newly discovered *MYB::TBC1D* fusion. **(B)** MRD kinetics at different treatment stages. **(C)** Kinetics of CAR-T copy number targeting CLL1 at different times after patient reinfusion.

A 10-year-old boy presented with a 1-week history of cough. Physical examination revealed petechiae on the anterior chest wall. Laboratory findings included leukopenia (WBC 1.1 × 10⁹/L), neutropenia (ANC 0.35 × 10⁹/L), anemia (Hb 68 g/L), and thrombocytopenia (PLT 85 × 10⁹/L). BM morphology suggested AML-M5 subtype. Immunophenotyping by MFC (HLA-DR, CD9, CD123, CD56, CD33, CD 36, CD15, CD7, CD4, and TDT+) and BM histopathology supported a diagnosis of myeloid leukemia. Molecular analysis identified a novel *MYB::TBC1D5* fusion (Figure 2A) and an *NRAS* mutation. Based on these findings, the patient was diagnosed with AML (intermediate risk) and treated with the CALSIII-AML18 (11) protocol, achieving complete remission (CR) after first induction therapy.

Eight months following diagnosis, he was readmitted with a 10-day history of testicular swelling. Examination revealed a 2-cm firm nodule on the frontal-parietal bone. BM morphology showed 49% myeloid blasts, and immunophenotyping detected 33.7% abnormal myeloid cells, indicating first relapse. MRI studies confirmed metastatic involvement: Skull MRI demonstrated subcutaneous nodules (left parietal and right maxillofacial regions), and testicular MRI revealed bilateral enlargement with abnormal signals, consistent with leukemic infiltration. The diagnosis was revised to relapsed AML with testicular, BM, and cutaneous involvement.

Given preferential CLL1 expression on AML stem cells, emerging evidence of anti-CLL1 CAR-T efficacy in relapsed/refractory pediatric AML, and the patient’s confirmed CLL1 expression by flow cytometric, he received fludarabine and cyclophosphamide preconditioning followed by anti-CLL1 CAR-T infusion following idarubicin bridging (12, 13). He developed fever on day 1 following CAR-T and grade 2 hypotension on day 4, managed with tocilizumab and fluid resuscitation (CRS grade 2 per ASTCT criteria). Post-CAR-T BM evaluation of MRD by MFC showed 2.1% level. Subsequent bridging therapy preceded allo-HSCT preparation, during which BM analysis confirmed secondary relapse with reclassification as BPDCN (HLA-DR, CD56, CD123, and CD304 +) rather than AML. MRD kinetics are shown in Figure 2B. At initial presentation, the blast morphology was highly monoblastic, and the immunophenotype, while positive for CD123, CD4, and CD56, also showed partial expression of CD33 and lacked definitive lineage-specific markers such as MPO, CD3, or CD19. In the context of monoblastic morphology, the diagnosis of acute myeloid leukemia (AML-M5) was favored, and the possibility of BPDCN was initially under-recognized. Upon central review at relapse, a more comprehensive immunophenotypic analysis, including strong coexpression of CD123, CD4, CD56, and CD304, with negativity for MPO and lysozyme, confirmed the diagnosis of BPDCN.

Following preconditioning, the patient received a combined graft from his uncle (5/10 HLA match) and umbilical cord blood (9/10 HLA match). Complete donor chimerism was achieved following transplantation, with subsequent MRD clearance. This complex clinical course underscores the critical importance of early, accurate diagnosis and the optimal timing for transplantation in BPDCN. Diagnosis should adhere to established guidelines (e.g., NCCN, French Network guidelines), necessitating expanded screening for DC-associated markers (e.g., CD123, TCF4, and CD303) in AML-suspected cases with DC-related molecules (14). While AML-type chemotherapy can induce initial complete remission in BPDCN, relapse remains common, and allo-HSCT may represent the sole curative option following relapse. Although CD123-targeted CAR-T is an emerging therapeutic strategy for BPDCN, this case demonstrates the feasibility of CLL1-directed CAR-T in achieving CR. CLL1 is highly and consistently expressed on the surface of BPDCN blasts. Upon binding to CLL1, CAR-T cells become activated, proliferate, and exert potent cytotoxic effects against BPDCN cells. However, potential challenges remain, including antigen escape (downregulation or loss of CLL1 expression under selective pressure) and limited *in vivo* persistence of CAR-T cells, both of which are important considerations for long-term efficacy. In this case, transient CAR-T persistence (Figure 2C) correlated with rapid MRD resurgence, highlighting the need for enhanced CAR-T durability. Preclinical evidence indicates that tandem CLL1/CD123 CAR-T cells exert potent cytotoxicity against CLL1 + CD123 + leukemia cell lines and primary AML blasts, warranting investigation of coexpressing BPDCN (15). This case demonstrates that bridging CAR-T therapy with allo-HSCT may help overcome these limitations by providing a sustained graft-versus-leukemia effect and immune reconstitution.

In summary, we report two pediatric cases of BPDCN with distinct therapeutic approaches. The first case achieved sustained complete remission through ALL-based chemotherapy (with intensified intrathecal therapy for rare CNS involvement) followed by allo-HSCT, concurrently providing the first documented comprehensive drug sensitivity profile of BPDCN cells. The second case was initially misdiagnosed as AML and harbored an *MYB::TBC1D5* rearrangement. To our knowledge, this *MYB::TBC1D5* rearrangement has not been previously reported in BPDCN. Given the higher frequency of *MYB* alterations in pediatric BPDCN, screening for *MYB* rearrangements, including via RNA sequencing, should be considered in the diagnostic workup of pediatric BPDCN, as it may have implications for risk stratification and the development of future targeted therapies. This patient experienced bone marrow and testicular relapse following AML chemotherapy, becoming the first reported BPDCN patient to attain CR2 with CLL1-targeted CAR-T therapy. While CLL1 is an emerging target in AML, its successful application in BPDCN is scarcely documented. Our case represents, to our knowledge, one of the first reports of CLL1-directed CAR-T therapy to achieve a second complete remission in a pediatric patient with relapsed/refractory BPDCN, followed by successful consolidation with allo-HSCT. However, transient CAR-T persistence preceded rapid recurrence, indicating that despite BPDCN's myeloid origin, the pathobiological relevance and therapeutic efficacy of CLL1 require further elucidation.

Our second case highlights the diagnostic challenge of pediatric BPDCN, which can be misdiagnosed as AML—particularly monoblastic leukemia—due to overlapping morphologic features and occasional coexpression of myeloid markers. This diagnostic pitfall is well documented, especially in pediatric settings where BPDCN is exceedingly rare. A high index of suspicion and systematic application of a diagnostic panel including CD123, CD4, CD56, CD303, and TCL1, with exclusionary markers, are essential. Our case underscores that the classical immunophenotypic triad, if present, should strongly point toward BPDCN even in the face of AML-like morphology, necessitating a definitive diagnosis to guide appropriate therapy. Early accurate diagnosis is paramount, and pediatric leukemia patients expressing CD36 and dendritic cell markers without classic myeloid markers (e.g., CD13, CD33, or MPO) should be considered BPDCN rather than AML.

Given the absence of standardized BPDCN therapy, ALL-based regimens may demonstrate superior efficacy compared with AML-type chemotherapy, despite BPDCN's myeloid classification in these individuals. CLL1-targeted CAR-T immunotherapy followed by allo-HSCT represents a promising novel strategy, while *in vitro* drug sensitivity profiling supports exploring combinatorial approaches incorporating BCL-2 inhibitors such as venetoclax. The management of pediatric BPDCN remains challenging due to its rarity and clinical heterogeneity. There is a pressing need for prospective, multicenter collaborative studies to establish standardized pediatric-specific protocols. Promising avenues include evaluating front-line tagraxofusp (16), integrating venetoclax-based regimens (9, 17), and developing CAR-T strategies to mitigate antigen escape (15). Clinical trials are underway and may provide much-needed evidence to guide future therapy.

## Data Availability

The raw data supporting the conclusions of this article will be made available by the authors, without undue reservation.

## References

[1] Cuglievan B Connors J He J Khazal S Yedururi S Dai J Blastic plasmacytoid dendritic cell neoplasm: a comprehensive review in pediatrics, adolescents, and young adults (AYA) and an update of novel therapies. Leukemia. (2023) 37(9):1767–78. 10.1038/s41375-023-01968-z 37452102 PMC10457206

[2] Sakamoto K Takeuchi K. Diagnostic approach to blastic plasmacytoid dendritic cell neoplasm: historical perspectives and current understanding. J Clin Exp Hematop. (2025) 65(1):1–16. 10.3960/jslrt.24069 40159280 PMC12051425

[3] Booth CAG Bouyssou JM Togami K Armand O Rivas HG Yan K BPDCN MYB fusions regulate cell cycle genes, impair differentiation, and induce myeloid-dendritic cell leukemia. JCI Insight. (2024) 9(24):e183889. 10.1172/jci.insight.183889 39499902 PMC11665559

[4] Pagano L Zinzani PL Pileri S Quaglino P Cuglievan B Berti E Unmet clinical needs and management recommendations for blastic plasmacytoid dendritic cell neoplasm: a consensus-based position paper from an ad hoc international expert panel. Hemasphere. (2023) 7(3):e841. 10.1097/HS9.0000000000000841 36844178 PMC9946418

[5] Cai T Gouble A Black KL Skwarska A Naqvi AS Taylor D Targeting CD123 in blastic plasmacytoid dendritic cell neoplasm using allogeneic anti-CD123 CAR T cells. Nat Commun. (2022) 13(1):2228. 10.1038/s41467-022-29669-8 35484100 PMC9051102

[6] He GQ Lei YP Huang DW Gao J Yang R. Philadelphia chromosome-like acute lymphoblastic leukemia with concomitant rearrangements of CRLF2 and ABL1: a pediatric case report. BMC Pediatr. (2024) 24(1):517. 10.1186/s12887-024-04991-w 39127642 PMC11316372

[7] Wang H Chan KYY Cheng CK Ng MHL Lee PY Cheng FWT Pharmacogenomic profiling of pediatric acute myeloid leukemia to identify therapeutic vulnerabilities and inform functional precision medicine. Blood Cancer Discov. (2022) 3(6):516–35. 10.1158/2643-3230.BCD-22-0011 35960210 PMC9894568

[8] Garnache-Ottou F Vidal C Biichlé S Renosi F Poret E Pagadoy M How should we diagnose and treat blastic plasmacytoid dendritic cell neoplasm patients? Blood Adv. (2019) 3(24):4238–51. 10.1182/bloodadvances.2019000647 31869411 PMC6929390

[9] Montero J Stephansky J Cai T Griffin GK Cabal-Hierro L Togami K Blastic plasmacytoid dendritic cell neoplasm is dependent on BCL2 and sensitive to venetoclax. Cancer Discov. (2017) 7(2):156–64. 10.1158/2159-8290.CD-16-0999 27986708 PMC5296248

[10] Kosasih HJ Healey G Brennan MS Bjelosevic S Sadras T Jalud FB A novel MYB::PAIP1 oncogenic fusion in pediatric blastic plasmacytoid dendritic cell neoplasm (BPDCN) is dependent on BCL2 expression and is sensitive to venetoclax. Hemasphere. (2024) 8(2):e1. 10.1002/hem3.1 38435422 PMC10878182

[11] Gao L Ju X Jiang H Liao N Wang N Zhai X Outcomes of children and adolescents with acute myeloid leukemia given a low-versus standard-dose chemotherapy regimen for remission induction (CALSIII-AML18): a multicenter, phase 3, randomized, noninferiority trial. Blood. (2023) 142(Supplement 1):729. 10.1182/blood-2023-189253

[12] Zhang H Bu C Peng Z Li G Zhou Z Ding W Characteristics of anti-CLL1 based CAR-T therapy for children with relapsed or refractory acute myeloid leukemia: the multi-center efficacy and safety interim analysis. Leukemia. (2022) 36(11):2596–604. 10.1038/s41375-022-01703-0 36151140

[13] Zhao Y Bai X Guo S Zhang X Liu J Zhao M Efficacy and safety of CAR-T therapy targeting CLL1 in patients with extramedullary diseases of acute myeloid leukemia. J Transl Med. (2024) 22(1):888. 10.1186/s12967-024-05705-7 39358720 PMC11446059

[14] Kharfan-Dabaja MA Lane AA Pemmaraju N. How I treat blastic plasmacytoid dendritic cell neoplasm. Blood. (2025) 145(6):567–76. 10.1182/blood.2024024262 39374520

[15] Wang XY Bian MR Lin GQ Yu L Zhang YM Wu DP. Tandem bispecific CD123/CLL-1 CAR-T cells exhibit specific cytolytic effector functions against human acute myeloid leukaemia. Eur J Haematol. (2024) 112(1):83–93. 10.1111/ejh.14104 37712633

[16] Pemmaraju N Sweet KL Stein AS Wang ES Rizzieri DA Vasu S Long-term benefits of tagraxofusp for patients with blastic plasmacytoid dendritic cell neoplasm. J Clin Oncol. (2022) 40(26):3032–6. 10.1200/JCO.22.00034 35820082 PMC9462530

[17] Khalife-Hachem S Pages A Akoury E Bonnet S Birsen R Busquet C Venetoclax-proteasome inhibitor-dexamethasone for unfit patients with blastic plasmacytoid dendritic cell neoplasm. Blood Adv. (2025) 9(4):793–6. 10.1182/bloodadvances.2024014590 39642332 PMC11869961

